# Go the extra mile

**DOI:** 10.1002/jhm.12986

**Published:** 2022-10-25

**Authors:** Blake Hardin

**Affiliations:** ^1^ Graduate Medical Education University of Michigan Medical School Michigan Ann Arbor USA

When I was 5 years old, I nearly lost my life due to complications from a previously unknown congenital birth defect. I came to be diagnosed with intestinal malrotation, resulting in midgut volvulus. I then underwent emergency surgery to remove most of my bowel, ultimately saving my life but causing short bowel syndrome. Beyond the medical jargon, my family and I, even at a young age, knew that I would be different from other kids and that the trajectory of my life would be altered for good. As I look back on my life thus far, 17 years later, only then can I start to look forward as I embark on my first step in my medical education in a few short months. I can remember how my healthcare team going the extra mile in my care made a difference in me pursuing medicine rather than fleeing from it.

When I was 5 years old, I was just an average kid finishing kindergarten. I enjoyed spending time with my family, running and jumping at playgrounds, and playing video games. I loved going to my grandparents' house when my parents were at work, and I would watch TV with them and have dart blaster fights. Life was simple. Nobody knew that I would soon be fighting for my life. The day after I graduated from kindergarten, my parents took me to my general pediatrician because I was extremely lethargic and had stomach pain, which my parents thought was from a stomach bug they had just gotten over. Upon examination, I was jaundiced, my eyes and skin appeared yellow, and my blood pressure was abnormally low. My pediatrician's demeanor turned from relaxed to calm but deeply concerned. She called an ambulance, and like a chaotic dance, there was a flurry of movement with nurses, medical assistants, and emergency medical services personnel all moving in lockstep to stabilize me and send me to the nearest hospital for evaluation.

As my parents describe it, everything was a blur. At the nearby hospital, the medical staff quickly realized that I required more specialized care fast. Nearly in a blink of an eye, I was placed into a medical helicopter and rushed to the nearest children's hospital while my parents watched their little boy fly through the air. Multiple surgeries removed most of my intestines and one medically induced coma later, I managed to pull through. Although this was not the end, I spent months admitted to the hospital while I recovered. I had a gastrostomy (G) tube implanted to supply supplemental nutrition that I now needed in addition to my oral diet. I watched as other children with similar conditions became my roommates, some of whom never left the hospital. While I remember some of this, I realize I have pushed much of what I felt and experienced into the recesses of my mind, never to be thought of again. Even today, when I discuss my time in the hospital with my family, they struggle to dwell on it due to the trauma that has left its mark to this day.

While the trauma left an enormous impact on my family and my life moving forward, I fully realize that the excellent care I received made an even more meaningful impact on us. As soon as I entered the hospital, my entire care team treated me not simply as a pathophysiology to be corrected or as a “zebra” to be studied. Instead, my family and I became a part of their family, treating us with dignity, compassion, and respect throughout my healthcare journey. Even at a young age, they explained my condition at a level I could understand and integrated my family and me into the care team. I vividly remember when I woke up from an operation, my surgeon made a set of makeshift Frankenstein “bolts” out of arts‐and‐crafts materials that he placed over my head, reminding me that even being hospital‐bound and medically complex, I am more than my illness; I was a kid first. Although there were moments when I was in such immense pain that neither morphine nor my mom could soothe me, I can remember the times I was deathly afraid of having my G‐tube replaced. The nurses and child life staff calmly reassured me and used pain‐management strategies to help me through the process. It is because of the compassionate, patient‐centered, and family‐centered care I received that the positive moments are the ones that stand out the most.

My journey with chronic illness is, as such, my own journey and does not reflect the diverse spectrum of experiences that others living with disabilities or chronic conditions face. I learned to treat my disease and lived experience not as a weakness but as one of my greatest strengths. I have been blessed with positive healthcare experiences throughout my life, those that made my family and I feel heard and seen, and those that made me feel like a human being, not a disease. However, I have heard horror stories from friends and loved ones navigating chronic illnesses, who now view the medical field as an agent of trauma and anguish rather than one of compassion and understanding. One such friend recalled their most recent torturous healthcare experience, pleading with me to remember her story and, as a future doctor, to “go the extra mile” for my patients.

To go the extra mile for patients, we must recognize that people with chronic illnesses and those requiring more complex, multidisciplinary care often fall victim to the weaknesses of the healthcare system. As healthcare providers, it is easy to fall into the trap of only considering the consequences of the care directly provided in the context of the patient's clinic or hospital visit. However, patients often require care from physicians of different specialties and healthcare professionals, such as nurses, physical therapists, pharmacists, dietitians, social workers, and more. Patients and families need all the professionals that are part of their care team to have open, honest, and transparent communication between them to ensure continuity of care and prevent them from slipping through the cracks. When healthcare professionals fail to communicate with each other effectively to coordinate care, the burden ultimately falls on the patient.

We must also recognize the importance of every word we say to patients during their hospital stay. Although healthcare team members interact with many patients daily and are knowledgeable of the language of medicine, every patient and family member comes with different knowledge and experience regarding healthcare. Regardless, patients and families in their most vulnerable states will hang onto every single word that members of the healthcare team use. Words can empower, inspire, and comfort, but they can also confuse, harm, and even traumatize. To this day, my family can remember my initial illness and the language my care team used. Although they did not mince words regarding the seriousness of my condition, they explained what was happening to me in a way they could understand and, with a calm demeanor, provided a small but powerful source of comfort they could draw from.

Patients with chronic illnesses also often struggle with mental health, which must be considered even in hospital medicine. Not to mention the burden of living with a condition that affects many aspects of a patient's life, a postdischarge care plan has the potential to interfere with the parts of one's life that give joy and meaning. Sometimes, the proposed care plan for a patient and their family may come at the expense of their mental health and could cause anxiety, stress, or depression. When a patient is discharged from the hospital, the care team only truly knows what happens within the bounds of the hospital. Outside, the care plan must work around the patient's life. Therefore, physicians must be flexible and develop a care plan coordinated with other health professionals involved in patient care and concordant with patients' contexts, experiences, values, and perspectives.

As I soon begin medical school at the institution that saved my life, a journey that has come full circle, I think about the question that has driven me throughout my life: what meaning can I draw from my chronic illness? The answer is simple: go the extra mile. Growing up “medically complex,” I can connect with my patients on a level few can. When I walk into a patient's room, I can listen to them, believe them, and show them that they matter. I can advocate on their behalf to ensure they receive the care I would expect for myself. I can dismantle the traditional physician‐patient hierarchy and treat them as my equal, acknowledging them as an expert in their own body. I can take the time to sit with them and educate them on the treatment plan we both agree on, facilitating open and transparent dialogue. With my positive healthcare experiences, rather than seeing it as traumatic, I can view it as a system with the potential for healing.

## CONFLICT OF INTEREST

The author declares no conflict of interest.

